# The shape of the human language-ready brain

**DOI:** 10.3389/fpsyg.2014.00282

**Published:** 2014-04-04

**Authors:** Cedric Boeckx, Antonio Benítez-Burraco

**Affiliations:** ^1^Catalan Institute for Advanced Studies and Research (ICREA)Barcelona, Spain; ^2^Department of Linguistics, Universitat de BarcelonaBarcelona, Spain; ^3^Department of Spanish Philology and its Didactics, University of HuelvaHuelva, Spain

**Keywords:** language-ready brain, cognitive biology, evolution of language, comparative neuroscience, human evolution, globularity, biolinguistics

## Abstract

Our core hypothesis is that the emergence of our species-specific language-ready brain ought to be understood in light of the developmental changes expressed at the levels of brain morphology and neural connectivity that occurred in our species after the split from Neanderthals–Denisovans and that gave us a more globular braincase configuration. In addition to changes at the cortical level, we hypothesize that the anatomical shift that led to globularity also entailed significant changes at the subcortical level. We claim that the functional consequences of such changes must also be taken into account to gain a fuller understanding of our linguistic capacity. Here we focus on the thalamus, which we argue is central to language and human cognition, as it modulates fronto-parietal activity. With this new neurobiological perspective in place, we examine its possible molecular basis. We construct a candidate gene set whose members are involved in the development and connectivity of the thalamus, in the evolution of the human head, and are known to give rise to language-associated cognitive disorders. We submit that the new gene candidate set opens up new windows into our understanding of the genetic basis of our linguistic capacity. Thus, our hypothesis aims at generating new testing grounds concerning core aspects of language ontogeny and phylogeny.

## HYPOTHESIS AND OVERVIEW

The aim of this paper is to contribute to the field of biolinguistics, here understood as an umbrella term encompassing all the interdisciplinary attempts to identify the biological foundations of our species’ ability to spontaneously develop mental rule systems that are put to use in thought and communication. Such rule systems, known as natural languages, have well-defined properties that decades of linguistic research have revealed and that, taken together, make these systems different from what other species are mentally and behaviorally capable of (Chomsky, 1965; Pinker, 1994; Boeckx, 2010). We endorse the conclusion that it is aspects of our biology, specifically of our brain, that endow us with this mental ability.

In the generative linguistics tradition, this biological endowment is referred to as “Universal Grammar” or the “Language Organ” (Chomsky, 1965, 1975). Because these terms have come to be seen as too ideologically loaded, we prefer to speak here of the “language-ready brain.” This term has been adopted by several researchers of very different theoretical persuasions (Kegl, 2004; Arbib, 2012), and it has several advantages over its competitors. First, the term draws attention to the brain as the focus of inquiry. Second, it enables us to keep clearly separate two entities: one, the language-ready brain, understood as the cluster of brain properties that sets the stage for language ontogeny and phylogeny, and the other, language, understood as the collection of properties that humans eventually acquire as a result of social interactions. As Deacon (2010) points out, building on differences between two songbirds, the White-backed Munia and its domesticated cousin, the Bengalese finch, documented by Okanoya (2004), behavioral complexity is likely to have important consequences at the level of brain organization. In the case of songbirds, the domesticated strain of the wild White-rumped Munia, the Bengalese finch, is known to have a distinct song pattern with a more complicated syntax than the wild strain. Interestingly, Wada et al. (2013) not only identified differential androgen receptor (AR) expression in basal ganglia nucleus Area X GABAergic neurons between the two strains, they also revealed an epigenetic modification: DNA methylation state in regions upstream of AR in Area X.

A similar state of affairs is likely to hold when we compare the language-ready brain and the fully linguistic brain. In the case of the latter, we expect epigenetic changes, as areas are recruited to enable vocalization of complex signals, reading, writing, and so on (what Dehaene, 2009 calls “neuronal recycling”).

Whereas the linguistic systems that the modern human brain internalizes depend, of course, on the brain being language-ready, it is clear that many properties of languages are also the products of cultural evolution (Deacon, 1997; Arbib, 2012; Okanoya, 2012). In others words, in order to eventually characterize modern linguistic systems completely, it will be necessary to appeal to a broad range of evolutionary mechanisms. In particular, it will be necessary to characterize adequately the emergence of the socio-cultural contexts that can support, enhance, and perhaps even select for the use of our linguistic capacity. Offering such a complete characterization of language evolution is not our goal here. It is a far too demanding task for any single paper. Our aim is more modest. We seek to shed light on the emergence of the language-ready brain understood as but one aspect of the fully fledged linguistic brain of modern humans.

Such a fully fledged linguistic brain crucially requires, in addition to those aspects we focus on below, a proper description of the externalization component necessary for cultural transmission, which has at its core the sensorimotor systems dedicated to speech for spoken languages and to signing for sign languages. This is the topic of much work, and rapid progress in current biolinguistics, which we will not review here. We refer interested readers to Jarvis (2004), Fitch (2010), Arbib (2012), and Morrill et al. (2012) for comprehensive treatments.

A complete understanding of the modern linguistic brain also requires hypotheses concerning the social conditions that facilitate the learning of cultural variants (Tomasello, 1999, 2008, 2009; Kirby, 2013). Covering all of these aspects would obviously take us too far afield. We focus on properties of the language-ready brain that we feel have so far been neglected, and which we hypothesize are central to language ontogeny and phylogeny. Thus, we ask readers to view our hypothesis as the identification of an additional piece of a larger puzzle, to be complemented with the existing literature on externalization and communication. To be perfectly explicit: although we do not address details of certain properties such as vocal learning, we do not mean to diminish the importance of these in characterizing our linguistic brain. We take the human language faculty to be similar to many other traits: a mosaic made up of various components of distinct evolutionary origins (see Boeckx, 2013a). The hypothesis we develop in this paper is intended to address a facet of this mosaic for which substantial gaps in our understanding remain to be filled, with few leading candidate hypotheses on offer.

The facet we focus on pertains to the syntax–semantics interface: the characteristic syntactic complexity of human language that gives rise to compositional meaning. While we recognize the possibility of an evolutionary continuum regarding syntactic abilities, we want to ask which aspect of our brain is responsible for the more advanced form of combinatorial syntax attributed to our species.

Building on Broca’s writings (see Harrington, 1987), it has often been hypothesized that lateralization patterns are central to characterize the language-ready brain (Crow, 2008). As reviewed in Toga and Thompson (2003), prominent asymmetries are indeed found in the gross anatomy of the two brain hemispheres in anatomically modern humans (AMHs). Noticeable protrusions of the hemispheres, anteriorly and posteriorly, are observed, as well as differences in the widths of the frontal and occipital lobes. These protrusions produce imprints on the inner skull surface, known as petalia. A twisting effect is also observed, known as Yakovlevian torque, in which structures surrounding the right Sylvian fissure are “torqued forward” relative to their counterparts on the left. The left occipital lobe is also splayed across the midline and skews the interhemispheric fissure in a rightward direction. A related shape asymmetry is also commonly observed in the occipital horns of the lateral ventricles: these tend to project more deeply into the occipital lobes on the left than on the right.

Although we believe that hemispheric asymmetries certainly play a role in characterizing linguistic competence at the brain level, at least two considerations convinced us that laterality cannot be as central as it is often taken to be. First, the distinctive pattern of lateralization observed in human adults appears to be acquired through linguistic interaction (Minagawa-Kawai et al., 2011). Second, brain laterality is an aspect of many species. It is salient, for example, in non-human vocal learners like birds (Moorman et al., 2012). Thus, to the extent that laterality bears on the linguistic brain, we think that it is likely to be tied to the communicative function of language, or what we have referred to above as the “externalization” component. We take the evidence coming from birdsong studies to be particularly suggestive in this regard. As reviewed in Berwick et al. (2011, 2012), birdsongs and human languages diverge mostly at the levels of syntax and semantics. Although songs display some syntactic rules and are not devoid of meaning, “there is no compelling evidence to date that birdsong matches the characteristic syntactic complexity of human language, arising from the composition of smaller forms like words and phrases into larger ones” (Berwick et al., 2012, p. 1), the type of syntax that linguists claim give rise to semantic compositionality. The similarities between birdsongs and human languages pertain to externalization. Given that we find lateralization patterns for the song circuit in birds, we think it reasonable to conclude that the asymmetries found in the human brain are not responsible for the syntax–semantics interface that we will focus on in what follows. This conclusion is in fact what Broca (1861) appears to have had in mind, since he clearly distinguished between the faculty of language and the faculty of articulate language. For Broca, only the latter was associated with lateralization patterns. Our conclusion is also in line with more recent studies casting doubt on a direct link between laterality and language as a whole (see, among others, Benítez-Burraco and Longa, 2012; Bishop, 2013; Cochet and Byrne, 2013; Fitch and Braccini, 2013; Gómez-Robles et al., 2013; Greve et al., 2013; Hancock and Bever, 2013).

Rather than laterality, we hypothesize that the relevant autapomorphy is one that has so far received no attention in the context of biolinguistics, and that is most visibly expressed in the globular aspect of the human endocranial morphology, particularly salient in early postnatal development (Vannucci et al., 2013). We will refer to this trait as “globularity” in what follows. As we will show in the next two sections, we have reasons to claim that the neuroanatomical and physiological properties giving rise to globularity contributed significantly to making our brain language-ready. Once we have made this clear, we will use the information to generate some testable predictions of our hypothesis. In particular, in Section “Molecular Basis,” we will put forward a set of candidate genes that contribute to the reliable emergence of a globular, language-ready brain and that could be used in future studies in the genetic basis of our linguistic ability.

## GLOBULARITY

A detailed examination of endocasts from fossil specimens of the genus *Homo* some 10 years ago (Bruner et al., 2003; Bruner, 2004) has revealed that modern humans, in contrast to the otherwise heavily encephalized Neanderthals, “show a species-specific neomorphic hypertrophy of the parietal volumes, leading to a dorsal growth and ventral flexion (convolution) and consequent globularity of the whole structure” (Bruner, 2004, p. 279). Subsequent research (Gunz et al., 2010, 2012; Neubauer et al., 2010; Lieberman, 2011) has established that globularity is the result of a unique developmental trajectory in modern humans, taking place at a stage of growth where the brain is the primary determinant of skull shape. (Incidentally, this very difference between Neanderthals and us argues against the idea, still popular in neuroscience, that globularity is merely a side-effect of upright walking in animals, given that Neanderthals and us had quite the same mode of locomotion).

Comparing endocranial shape changes during ontogeny in humans and chimpanzees, Neubauer et al. (2010) have shown that “while some aspects of the pattern of endocranial shape change are shared between humans and chimpanzees, the shape trajectories differ substantially directly after birth until the eruption of the deciduous dentition: in humans but not in chimpanzees, the parietal and cerebellar regions expand relatively (contributing to neurocranial globularity) and the cranial base flexes within the first postnatal year when brain growth rates are high.” (p. 555). Neubauer et al. (2010) refer to this early developmental stage as the “globularization phase,” but we will continue to use the term “globularity” to refer to both the developmental process and to the end product of this process.

Neubauer et al. (2010) stress that the shape changes giving rise to globularity are unique to humans and do not occur in chimpanzees before or after birth. Nor do they occur in Neanderthals (Gunz et al., 2010, 2012). Although Neanderthals had brain sizes comparable to modern humans, their brain cases were elongated and not globular. Comparing shapes of virtual endocasts extracted from computed-tomographic scans of crania of modern humans and virtual reconstructions of fossil humans, including the Neanderthal neonate Le Moustier 2 and Mezmaiskaya, Gunz et al. (2010, 2012) conclude that the globularization phase seen in the neurocranial development of modern humans after birth is absent from Neanderthals, confirming Bruner et al.’s (2003) claim that modern humans and Neanderthals reached large brain sizes along different evolutionary pathways.

In sum, modern paleoneurology tells us that compared to our closest living and extinct relatives, humans have a large, specialized, and complex brain embedded in a uniquely shaped braincase. Specifically, the research we draw from in this section associates the emergence of this novel morphological trait with a distinctive developmental trajectory at the level of the brain.

As is well-known, brains do not fossilize, and only indirect evidence from fossil endocasts, combined with evidence from modern humans and our closest living relatives, the great apes, is what one has to rely on. But we are confident about the inferences about brains drawn in the literature we have mentioned in this section, for all the reasons reviewed in Zollikofer and Ponce de León (2013).

Along with the authors of the works just reviewed, we take it to be reasonable to think that the morphological changes giving rise to globularity are the products of factors that have important neurofunctional consequences. In other words, globularity is not just a superficial property of braincases. It crucially entails modifications of neural connections, for it is brain growth that influences the formation and shape of the braincase, especially in the first year of life. As we will see in Section “Molecular Basis,” all the genes that we have been able to link to globularity contribute significantly to neurogenesis, arealization of the neocortex, synaptic plasticity, and the like. In other words, they are not confined to bone formation. Indeed, the very signals they send to build the brain case are those that have been independently argued to contribute to brain organization. Thus, a crucial component of our hypothesis is that if the brain grows differently, it wires differently. Obviously, the differences are to be understood amidst the many commonalities that we expect to find in the context of encephalization. But, as we review in more detail below, even subtle changes can have wide-ranging implications for cognition. What we find particularly intriguing is that certain cognitive disorders known to result from deviations in neural connectivity also lead to deviations from the norm in the context of head shape, suggesting that there is indeed a link to explore between how the brain grows and how the head develops as a whole (see, e.g., Cheung et al., 2011 in the context of autism). In addition, differential growth is likely to lead to a reallocation of brain resources, or rewiring that may give rise to distinct cognitive phenotypes.

In the context of globularity, the results reported so far lead to a change of perspective in thinking about what makes the modern human brain special. In particular, it suggests a possible link between a special head shape and special aspects of our cognition. This is the link we want to explore. More precisely, we want to examine the possibility that globularity is what underlies our species’ language-readiness.

We thus assume, along with many authors, that Neanderthals’ brains were not language-ready, at least not in the way or to the extent in which sapiens’ brains are. This, of course, does not mean that Neanderthals did not engage in symbolic activities, or were incapable of vocal learning, or had no syntactic abilities at all. We certainly appreciate the range of anatomical evidence suggesting that Neanderthals had complex auditory and articulatory capacities not unlike ours (Martínez et al., 2004; D’Anastasio et al., 2013), and engaged in complex, symbolic, cultural practices (Zilhão et al., 2010; Rendu et al., 2014), some of which indeed used to be claimed to be unique to us. It is true that, while these abilities and practices were thought to be attested only in modern human populations, they were claimed to be closely linked to language, but such links were poor (Balari et al., 2011). As impressive as the Neanderthal achievements may be, we think it fair to conclude that as of now, “no data or analytical tools currently available” indicate that Neanderthals were “capable of the critical thought and syntactical ability necessary for complex language” (D’Anastasio et al., 2013, p. 6). Attempts to show otherwise (e.g., Dediu and Levinson, 2013) are inconclusive (Benítez-Burraco and Barceló-Coblijn, 2013; Berwick et al., 2013b), and a range of considerations continue to provide evidence for key cognitive differences between Neanderthals and AMHs (Wynn and Coolidge, 2011; Longa, 2013), differences that we will associate with the syntax–semantics interface in Section “Globularity and the Language-Ready Brain.”

In concluding this section, we would like to make two more remarks concerning globularity in connections with issues that have been frequently discussed in the neurolinguistic literature. In addition to moving us away from laterality, globularity suggests that not only brain size, but also shape matters. The size factor, understood as body/brain ratio, cannot, of course, be ignored. As reviewed in Deacon (1997), the brain of modern humans is an evolutionary and developmental outlier. At birth, it has the size of an adult chimpanzee brain and expands by a factor of 2 during the first postnatal year. Large neonatal brain size and rapid initial growth contrast with slow maturation, which extends well into adolescence. These aspects of the human brain undoubtedly play an important role in the emergence of modern human cognition. But we believe that they are not the whole story. Consistent with this stance, we expect cognitive innovations linked to brain size alone to be present in other hominins. That is to say, to understand traits uniquely associated with AMHs, we hypothesize that it is necessary to look beyond brain size.

In addition, globularity de-emphasizes the role of the frontal lobes in giving rise to modern human cognition. One of the most pervasive assumptions about human brain evolution has indeed been that it involved relative enlargement of the frontal lobes. The literature on globularity indicates that at the very least parietal volumes are equally important. As Bruner (2010) observes, “as brain size increases, the parietal lobes undergo relative flattening in non-modern humans. This pattern is stressed in Neanderthals, which show, however, a certain widening of the parietal volumes. Only *Homo sapiens* shows a generalized enlargement of the entire parietal surface.” (p. S77). It is indeed reasonable to think that the morphological changes in the parietal region are to be related to important neurofunctional consequences, complementing the functions of the frontal lobes.

In this context, it is worth taking seriously studies like Barton and Venditti (2013) or Smaers and Soligo (2013) showing that the size of human frontal lobes, and of specific frontal regions, is as expected relative to the size of other brain structures. Thus, although Barton and Venditti (2013) confirmed that absolute and proportional frontal region size increased rapidly in humans, this change was tightly correlated with corresponding size increases in other areas and whole brain size, and with decreases in frontal neuron densities. Barton and Venditti (2013) conclude that “the search for the neural basis of human cognitive uniqueness should therefore focus less on the frontal lobes in isolation and more on distributed neural networks” (p. 9001) Recent work on cognitive impairments essentially reaches the same conclusion (Turken and Dronkers, 2011; Dick and Tremblay, 2012). As will become evident in the next section, our position agrees with this perspective, which we think is gradually becoming the norm in neurolinguistics.

Having described the nature and origin of globularity, as well as the limits of hypotheses based on laterality and brain size, we are now in a position to formulate our hypothesis, which is to link globularity with the language-ready brain.

## GLOBULARITY AND THE LANGUAGE-READY BRAIN

As we saw in the previous section, we take it that globularity is not just a superficial property of braincases. It crucially entails modifications of neural connections. We wish to put forward the idea that the developmental trajectory giving rise to globularity is critical to the formation of a network of neural connections capable of supporting the most distinctive mode of cognition that numerous scholars have associated with language and that current evidence suggests is absent in Neanderthals. Put succinctly, the globular brain gives rise to the language-ready brain. Spelling out this hypothesis is the purpose of this section.

To be testable, our hypothesis requires us to articulate an explicit linking hypothesis between mind and brain, that is, between the properties we as linguists associate with language-readiness and the neural connections that could support such mental properties. Once this is done, we must show how these neural connections become available in the context of globularity.

Our hypothesis is that the species-specific anatomical component we have highlighted in the previous section is responsible for what is computationally unique about our species’ linguistic abilities. Thus, in order to link globularity to computational operations, we must first be clear about what is computationally unique about our mental life. In line with the recommendations formulated in Fitch (2009) and Poeppel (2005, 2011, 2012), we seek to formulate these computational properties “at a fine enough grain that one can discuss algorithmic and implementational approaches to [them]” (Fitch, 2009, p. 298). These computational properties should be, “ideally, elemental and generic…. Generic formal operations at this level of abstraction can form the basis for more complex linguistic representation and computation.” (Poeppel, 2005, p. 11).

Comparative psychology has established that unlike other species, modern humans excel at unifying and combining conceptual units that belong to distinct “core knowledge systems” (Spelke, 1994, 2000, 2004; Boeckx, 2010). Core knowledge systems roughly correspond to the well-known Fodorian “modules” (Fodor, 1983). They are the building blocks that enable animals to make sense of the world around them. As reviewed in Kinzler and Spelke (2007), we have very robust evidence for four or five core knowledge systems in many species: one system specializing in objects and their mechanical interactions, another specializing in agents (animate things) and their goal-directed actions, a third concerned with sets and numbers (number sense), a fourth dealing with places and geometric relationships (natural geometry), and a fifth core knowledge system dealing with social partners, groups, and relations, and the way we understand other minds (theory of mind). Core knowledge systems are at the root of our capacity to form rudimentary theories of the world around us. These theories are the foundations of physics (object mechanics), mathematics (number sense), biology (animate vs. inanimate beings), navigation (natural geometry), and psychology/social science (theory of mind). These core knowledge systems give us and other animals an intuitive grasp of what is going on in each of these domains.

There is a lot of evidence from a range of fields that humans are unique – or, to put it in the context of an evolutionary continuum, far better than other species – in transcending the signature limits of core knowledge systems, going beyond modular boundaries (Mithen, 1996; Carruthers, 2002, 2006; Spelke, 2003; Wynn and Coolidge, 2004; Pietroski, 2007; Hauser, 2009; Boeckx, 2011a,b). This ability, which has all the characteristics of a phase transition, is at the heart of cognitive novelty, and subsequently, material and cultural innovation, leading to the establishment of a new cognitive phenotype (Balari and Lorenzo, 2013; Boeckx, 2013a). This ability is what Hauser (2009) dubbed “humaniqueness.” Hauser (2009) defines the latter as follows: the ability to “create and easily understand symbolic representations of computation and sensory input,” to “apply the same rule or solution to one problem to a different and new situation,” and to “combine and recombine different types of information and knowledge in order to gain new understanding.”

Several of the authors just cited have put forth the idea that this distinctively human mode of thought is likely to be intimately related to language. We propose to capture this in the following way.

The core combinatorial operation in natural language that combines elementary linguistic units is called “Merge” in the terminology of Chomsky (1995), and it is the best candidate we know of to account for the combinatorial property at issue. According to Berwick et al.’s (2012) careful comparison between humans and song birds, the unrestricted combinatorial operator that Chomsky called Merge is absent in birds. Its absence means that bird songs are devoid of the compositional, freely combining, systematic, cross-modular semantics that is manifest in all human languages.

To be useful at all in thought and action, such a freely combining Merge must be regulated. As reviewed in Boeckx (2013a,b), we have linguistic reasons to believe that this regulation takes the form of integration/embedding: Merge is constrained in virtue of its interfacing with and being embedded inside cognitive systems responsible for interpretation and externalization. This regulation is what the formal linguistics literature refers to as “Spell Out” or “Unify” (Jackendoff, 2002; Hagoort, 2005). We suggest that this embedding takes the form of a generic coding mechanism that is already well established in neuroscience (Lisman, 2005; Buzsaki, 2008): internally generated oscillations at a high frequency such as the gamma range are embedded inside an oscillation operating at a lower frequency such as the alpha range. Such lower-frequency oscillations, characteristic of the thalamus, are known to be particularly well-suited to synchronize distant cortical areas (Whitman et al., 2013). Building on Boeckx (2013a,b), we hypothesize that this distant synchronization allows for the binding of features distributed across core knowledge systems.

The mechanism of achieving interareal communication via an adaptive coupling of rhythms synchronizing spatially distributed oscillations is a generic strategy of the brain, neither specific to humans nor to language. But we put forth the hypothesis that this mechanism gained its linguistic specificity and characteristic complexity when it found itself in a new anatomical context in our lineage: globularity.

As should be obvious from our discussion of what globularity is in Section “Globularity,” the new anatomical context that gave rise to the language-readiness does not refer to a specific brain area. Rather, it refers to a set of areas brought into connection with one another, a situation we may refer to as one of “dynamic connectivity.” Certainly, the prefrontal and parietal areas are involved, as these gained special prominence in a globular context, but we believe that in addition to these, there is at least a third anatomical structure that is traditionally ignored, but that we think is equally relevant to link globularity to language-readiness: the thalamus. This is the reason why we focus mainly on this brain structure here, returning to the contribution of the frontal lobe and the parietal lobe toward the end of the section, in the context of a fronto-parieto-thalamic network.

We have several reasons to adduce in support of our hypothesis concerning the relevance of the thalamus in the context of the globular and the language-ready brain.

First, the thalamus is central in more than one way. In a globular context, it sits right in the middle of the brain, and as such appears strategically placed to connect distant areas. As a matter of fact, it has been suggested that the globular brain shape of modern humans might have a positive effect on the wiring efficiency of the brain’s neural network (Hofman, 1989; Chklovskii and Stevens, 2000; McCarthy, 2001; Chklovskii et al., 2002). Developmentally, the thalamus forms from the diencephalon, and the cerebrum forms from the telencephalon. The telencephalon corresponds to the most bulbous part of the rostral end of the ballooning neural tube during development, and the diencephalon corresponds to the swelling just caudal to that. As the brain develops the cerebrum and cerebellum come to surround the thalamus. The thalamus has significant connections to them, so it’s sensible that it occupies a central position.

Second, Bishop et al. (2000), Price et al. (2006), and Chou et al. (2013) show that input from the thalamus, the main switching station in the brain for sensory information, is crucially required to complement the action of the genes in determining how the cerebral cortex grows into separate functional areas and subsequently dedicates itself to higher-order cognitive functions.

Third, the thalamus acts as a necessary relay center to connect many brain structures that have already been implicated in research on language (Lieberman, 2002; Murdoch, 2010): interactions between cortical areas and the basal ganglia or between cortical areas and with the cerebellum cannot take place in the absence of the thalamus (the same holds of the amygdala and other limbic structures that have been implicated in certain aspects of human “distinctness”). In fact, the literature on FOXP2 and its interactome has often mentioned the thalamus as an important expression site of the genes involved (Vargha-Khadem et al., 2005; Reimers-Kipping et al., 2011), a point to which we return in the context of molecular considerations in Section “Molecular Basis.”

Fourth, despite the cortical focus of many imaging studies and the technical difficulties in getting recordings from the thalamus, this brain structure’s role has been highlighted in some neurolinguistic studies, especially those pertaining to the syntax–semantics interface, the language component that is missing in non-human vocal learners (Wahl et al., 2008; David et al., 2011).

Fifth, there is rapidly accumulating evidence that cognitive disorders that are routinely associated with language and the distinctive mode of thought it entails such as schizophrenia, autism, dementia, major depression, verbal working memory impairments, etc. crucially involve thalamic disorders, especially as they affect the mediodorsal nucleus and the pulvinar. This is a complex topic which we hope to return to in future work. For now, let us just refer to important studies such as Parnaudeau et al. (2013) and works along similar lines (Popken et al., 2000; Byne et al., 2001; Dagenbach et al., 2001; Young et al., 2004; Alelu-Paz and Giménez-Amaya, 2008; Kovacs et al., 2013; Nair et al., 2013; Uhlhaas et al., 2013).

Finally, and perhaps most importantly, outside of language proper, the thalamus has routinely been assigned a key role in controlling attention, regulating oscillations generated in the cortex, etc. (Saalmann et al., 2012) – functions that, though not specific to language, must surely also be part of a comprehensive neural characterization of the language-ready brain.

Many neuroscientists continue to think of the thalamus simply as a relay station, where sensory information from the periphery converges and is then passed on to the cortex. The cortex is thought to be the site of perception and cognition, with different cortical areas specialized to subserve different functions. Communication between cortical areas can be mediated by axonal tracts running in the white matter of the cortex. This leads readily to the view that once information reaches the cortex it is processed and integrated with other information about the external world and internal states entirely within the cortex, resulting in conscious perception or some kind of motor or emotional output. But Theyel et al. (2009) demonstrate unequivocally that cortical areas can also pass information indirectly via the thalamus.

It has been known for some time that communication between thalamus and cortex is bidirectional. According to Theyel et al. (2009) the thalamus receives, in fact, far more inputs from the cortex than it does from the periphery. As they note, the circuits between thalamus and cortex can be broken down into two main types: those that drive the activity of their target neurons (whether in thalamus or cortex) and those that act more to modulate the activity of their targets, especially their temporal responsiveness. These pathways can be distinguished based on their neurochemical profiles, the types of synapses that they form and, in the case of projections from thalamus to cortex, the layers which they innervate. Driving connections from thalamus project with quite precise topography to layers 4 and 6, while modulatory connections project more diffusely within layers 1 and 5. These modulatory connections from the thalamus are essential mediators of communication between cortical areas, due to their crucial role in the synchronization of ongoing neuronal oscillations.

As Theyel et al. (2009) note, this frequency tuning can be mediated by corticothalamocortical loops, where the corticothalamic connection is driving and the thalamocortical connection is modulatory. In this context, however, the information itself is transferred via direct cortical connections. Theyel et al. (2009) show that even if these cortical connections are severed, information can still be transferred from one cortical area to another if corticothalamocortical circuits remain intact. In this case both the corticothalamic and the thalamocortical connections are driving. This finding reinforces the important point that the function of the cortex cannot be divorced from that of the thalamus. It emphasizes that perception is not simply a matter of passing information along a hierarchy of processing stations. Rather, it is a process of reiterative comparison of top-down predictions with bottom-up information, much of which may be mediated by reverberating activity in corticothalamocortical circuits.

In his recent review on cortical dynamics, Singer (2013) strengthens our claim regarding the relevance of the thalamus, as he notes that thalamic input crucially allows for an enrichment of the range of oscillatory activity in different frequency bands (see also Cannon et al., 2014; Parnaudeau et al., 2013; Uhlhaas et al., 2013).

The modulatory or regulatory role of the thalamus is further enhanced when the thalamic reticular nucleus is taken into account. The thalamic reticular nucleus consists of a thin layer of GABAergic cells adjacent to the relay nuclei of the dorsal thalamus. It occupies a striking control position in the brain, sending inhibitory axons back to the thalamus, roughly to the same region where they receive afferents, and has been hypothesized to play a pivotal role in dynamic attention by controlling thalamocortical synchronization (Crick, 1984; Min, 2010).

Addressing the issue of the evolution of intelligence, Kircher and Glendenning (2002) point out that in addition to the size of the neocortex, the amount of neural inhibition to which the cortex is subjected may play a major role. As we have argued in the context of Merge, where we noted that a completely unrestricted Merge operation is cognitively unhelpful, and therefore requires embedding, Kircher and Glendenning (2002) observe that an expanded brain that is out of control is not helpful. There must be modulation of this enhanced cortex. Kircher and Glendenning (2002) show that a primary source of this modulation comes from the enhanced inhibitory capabilities of the thalamus, and the increased number of neurons sensitive to the most common inhibitory neurotransmitter found, GABA. By its influence on our neocortex, the thalamus provides greater control of neural processing. Kircher and Glendenning (2002) propose that it may be our ability to inhibit our cortex that has resulted in our increased “intelligence,” which many authors have linked to language for decades.

The range of evidence reviewed so far suggests to us that a proper characterization of the language-ready brain that does not recognize a central role to the thalamus is unlikely to be correct, for it would miss the critical engagement of the thalamus in regulating cortical activity. By providing low-frequency oscillations capable of embedding higher-frequency oscillations across distant brain regions, the thalamus provides the crucial regulation needed to form the sort of meaningful cross-modular conceptual structures that are characteristic of language.

In hindsight, it is somewhat surprising that the role of the thalamus is not yet well established in the neurolinguistic literature, despite the fact that the thalamus has been implicated in the context of many human-specific traits like intelligence or consciousness, which Darwin (1871) already suggested depend on the exercise of the language faculty. This is true even in models that go beyond the standard cortico-centric perspective on higher-order cognition (Lieberman, 2002). That globularity offers us independent reasons to focus on the thalamus suggests to us that our initial hypothesis can lead to some productive rethinking in this area. Hopefully, our hypothesis will help redirect attention to cases of thalamic aphasia, which have been known for a while even if their significance has tended to remain at the periphery of neurolinguistic models. Significantly, Crosson (2013), Hebb and Ojemann (2013), and Klostermann et al. (2013) review and re-assess the significance of thalamic aphasia and reach conclusions that go in the direction of our hypothesis. Our hypothesis may also help us re-assess the role of the thalamus in other aspects of our language-faculty, such as vocal learning, where the relevance of the thalamus has long been recognized (Jarvis, 2004; Person and Perkel, 2005), and recently re-emphasized (Goldberg and Fee, 2011, 2012).

Still, for all our emphasis on the thalamus, we do not want to leave the reader with the impression that this is the only relevant brain structure to link globularity and language-readiness. As should be clear, the thalamus gains its significance in the context of a network that involves the frontal and the parietal lobes.

Bruner (2004, 2010) already drew attention to these two lobes in the context of globularity, although he did not make the connection with language hypothesized here. Other works on fronto-parietal connections clearly converge with aspects of our hypotheses, even if they do not always recognize the role of the thalamus, or link them to language. For example, the function of the fronto-parieto-thalamic network envisaged here share properties with a family of models of higher-order human cognition such as the models formulated by Dehaene et al. (1998) and Tononi and Edelman (1998) in the domain of consciousness, the multiple-demand system of Duncan (2010, 2013), the “connective core” model of Shanahan (2012), or the integrative architecture for general intelligence and executive function in Barbey et al. (2012). These models recognize a crucial role for the fronto-parietal regions in achieving what we have referred to as cross-modular concept formation above, which we take to be the central aspect of language-readiness.

Thus, Dehaene et al.’s (1998) neuronal workspace model emphasizes the role of distributed neurons with long-distance connections, particularly dense in prefrontal, cingulate, and parietal regions, interconnecting multiple specialized, modular processors and “broadcasting” signals at the brain scale in a spontaneous and sudden manner, forming a “global neuronal workspace.” Through this workspace, Dehaene et al. (1998) claim that modular processors can exchange information very flexibly, that information can be accumulated across time and across different processors, that incoming information arising from analog statistical inputs can be discretized, and that chains of operations can be performed.

Already a century ago Ramón y Cajal (1909) had underlined the special morphology of the pyramidal cells from the cerebral cortex and suggested they might be the “substratum of the highest nervous activities.” Building on this insight, Dehaene et al. (1998) view as key building blocks of the workspace “a distributed set of cortical neurons characterized by their ability to receive from and send back to homologous neurons in other cortical areas, horizontal projections through long-range excitatory axons.” (p. 14529). As they point out, “long-range corticocortical tangential connections, including callosal connections, mostly originate from the pyramidal cells of layers 2 and 3” (p. 14529), and propose that “the extent to which a given brain area contributes to the global workspace would be simply related to the fraction of its pyramidal neurons contributing to layers 2 and 3, which is particularly elevated in […] dorsolateral prefrontal and […] inferior parietal cortical structures.” (p. 14529). These are, of course, particularly relevant regions in the context of globularity.

As Dehaene et al. (1998) note, the pyramidal neurons from layers 2 and 3 “establish, in addition, vertical and reciprocal connections with layer 5 neurons and thus corresponding thalamic nuclei. These connections contribute to both the stability and the dynamics of workspace activity, via, for instance, self-sustained circuits, but also mediate the direct access to and from the processing networks.” It is these connections with the thalamus that we believe are crucial to regulate the activity of long-distance cortical connections, leading to cross-modularity.

It is also worth pointing out that the fronto-parieto-thalamic network that we take to emerge in the context of globularity and to underlie the human brain’s language-readiness shares features of the top-down, fronto-parietal attentional regulation network (Miller and Buschman, 2013). It is a circuit that has been claimed to have evolved from the foraging network of primates and eventually came to be used in the context of foresight (Genovesio et al., 2014). The network we envisage also bears a family resemblance with the default mode network that Gruberger et al. (2011) claim is responsible for mind-wandering and inner speech, a function that Chomsky (2012) describes as more central to language than its communicative use. The network we envisage comes closest to what Vincent et al. (2008) call the “frontoparietal control system,” a network that is anatomically interposed between the dorsal attention system and the hippocampal–cortical memory system. The frontoparietal control system is said to be “uniquely positioned to integrate information coming from the other two systems and to adjudicate between potentially competing inner- vs. outer-directed processes” (p. 3334). The only missing component of these existing models is the thalamus. (An important exception is Bohlken et al., 2013, where the thalamus receives the attention that we think it deserves).

There may have been other benefits of an improved fronto-parietal network, regulated by the thalamus. According to a DTI analysis by Hecht et al. (2013), there is an increase in the ratio fronto-parietal vs. fronto-temporal connectivity from monkeys to apes to modern humans, which is a possible substrate for the evolutionary shift from emulation to imitation. Emulation here refers to the ability to copy the final product of an action, while imitation refers to the ability to copy a process. It is imitation that is likely to underlie the possibility of cultural innovation that is so characteristic of modern humans, as compared to our closest living relatives or even Neanderthals, to judge from the fossil record.

A recent study by Pearce et al. (2013) may give us some clue as to how the fronto-parieto-thalamic network invoked here may have achieved its degree of robustness in modern humans. Focusing on the fact that Neanderthals had larger eyes than our species, Pearce et al. (2013) suggest that more of their brain was devoted to seeing in the long, dark nights in Europe, at the expense of high-level processing. This is so because larger eyes entail a much larger visual processing area at the back of their brains. In other words, more of the Neanderthal brain would have been dedicated to vision and body control. A reduction of the visual area in modern humans has been independently supported by Sherwood et al. (2008), and it may have led to an expansion of the parietal region, and a re-allocation of the computational power of the pulvinar, the part of the dorsal thalamus that modulates cortical visual processing (Saalmann et al., 2012), in service of other cognitive domains, such as language. A recent study on ultra-fast speech comprehension in blind subjects (Dietrich et al., 2013) and another on language processing in congenitally blind adults (Bedny et al., 2011) also indicate a significant recruitment of the pulvinar.

In this respect, it is worth mentioning that Streidter (2005) reports that the pulvinar is disproportionally large in humans, compared to other nuclei that lack prefrontal connections. (This is true also of the mediodorsal nucleus.) Streidter (2005) goes on (p. 331f) to note that “the human pulvinar is especially intriguing because its enlargement is causally related to a major change in its embryogenesis. Only in humans does the pulvinar contain neurons that migrated into the thalamus from the telencephalon […] The other fascinating aspect of human pulvinar hypertrophy is that it involves mainly the dorsal pulvinar, which has strong reciprocal connections with the lateral prefrontal, parietal, and temporal cortices (refs. omitted). This dorsal pulvinar is probably unique to primates, and separate from the ventral pulvinar, whose major function is to convey visual information from the midbrain to the telencephalon. Collectively, these data indicate that what enlarged in humans is not a motley group of areas and nuclei, but an entire circuit that includes the lateral prefrontal cortex and several “associates” in both the neocortex and the thalamus.” In the same context, it is worth pointing out that Bruner et al. (2010) found a positive correlation between the parietal expansion that contributed to globularity and the morphology of posterior subcortical landmarks, including the thalamus.

Based on the evidence we have obtained from the literature, we hypothesize that the dorsal thalamus, specifically the pulvinar and the mediodorsal nucleus, played a significant role, but we recognize that only future progress in neurolinguistics will enable us to draw a more precise map of which parts of the thalamus are critical for language-readiness.

**FIGURE 1:**
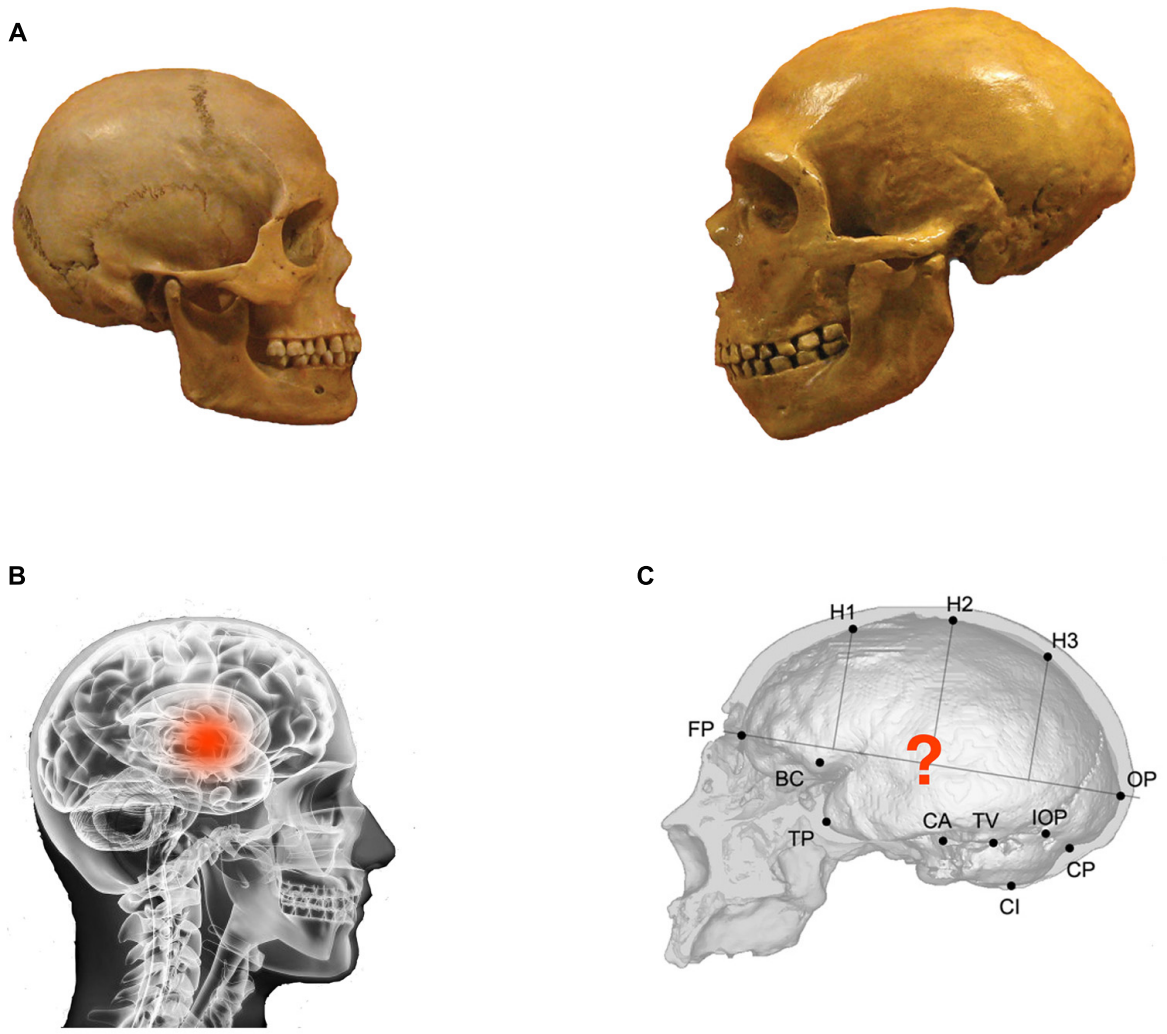
**Illustration of the hypothesis. (A)** Observable skull differences between anatomically modern human (left) and Neanderthal (right). **(B)** Identification of the strategic position of the thalamus in a modern human brain. **(C)** Representation of the hypothesis concerning the global connective role of the thalamus in an evolutionary perspective (image adapted from Bruner and Manzi, 2008).

To sum up this section (see **Figure 1**), our perspective on the emergence of the language-ready brain converges with much recent work in neuroscience concerning cognitive specialization, well captured in the following passage from Barton and Venditti (2013): “coordinated expansion of functionally and anatomically connected areas, potentially including both cortical and non-cortical regions.” As they note, and as we have just discussed, “neocortex, cerebellum, and intermediate nuclei, for example, show closely correlated evolution in terms of both volume and neuron numbers, after controlling for variability in the size or neuron numbers of other brain regions.” For Barton and Venditti (2013), “the evolution of frontal regions such as PFC [prefrontal cortex] may be best understood in terms of their participation in more distributed networks” “natural selection selectively enlarged such distributed networks and that these – rather than more localized size change of frontal cortical regions – are likely to form the basis of human cognitive specialization” (p. 9005).

## MOLECULAR BASIS

One of the major aims of biolinguistics is to arrive at a genetic characterization of language. If our hypothesis in Section “Globularity and the Language-Ready Brain” is on the right track, insight into the molecular basis of globularity is central to any ultimate genetic description of our linguistic competence. The goal of this section is to use our hypothesis to generate a set of candidate genes that will complement what can already be found in the literature on the genetics of language.

**FIGURE 2:**
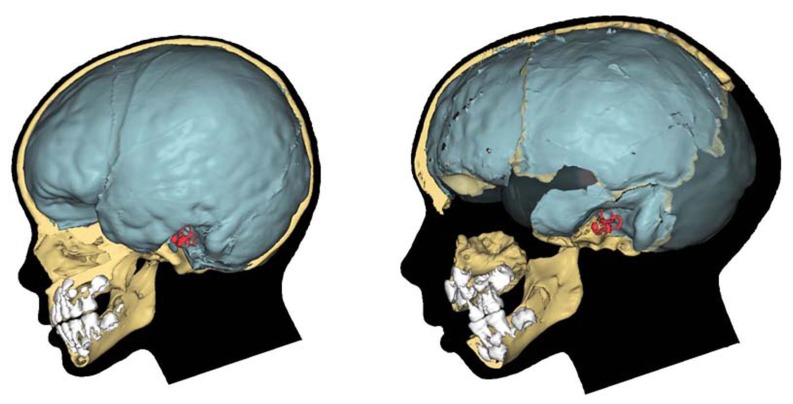
**Early brain shape comparison.** A modern human child (left) and the Gibraltar 1 Neanderthal (right; reproduced from http://www.aim.uzh.ch/morpho/wiki/CAP/N2).

Little is known about the molecular basis of globularity. As reviewed in Section “Globularity,” we know that it is a derived feature – indeed, a defining characteristic – of AMHs. We also know that it arises within the first year of life, when only modern human endocasts change rapidly from an elongated to a more globular shape (see **Figure 2**).

While trying to identify the molecular basis of our brain’s language-readiness, it is important to bear in mind that both the anatomical configuration of the brain and neural connections are not solely genetically controlled. Neural interconnection patterns become fixed only after birth in response to environmental stimuli (Zembrzycki et al., 2013). This means that not only genetic, but also epigenetic considerations must guide our search. In fact, we expect more differences in gene splicing patterns or in gene expression levels than in gene sequences. Having said this, at the genetic level, some differences still exist between the AMH and the Neanderthal–Denisovan genomes, with AMHs showing the derived variants and Neanderthals–Denisovans exhibiting the ancestral alleles (Green et al., 2010; Meyer et al., 2012). These genes probably act as stabilizers that reliably give rise to a globular braincase, and, if we are right, to a language-ready brain.

In the wake of the genomic revolution, extensive research has been done addressing the evolutionary trajectories of several sets of genes: (i) genes that have been shown to have a direct effect on some aspects of language, such as *FOXP2* (Krause et al., 2007); (ii) genes that control brain size, such as *ASPM* and *MCPH1* (Zhang, 2003; Montgomery et al., 2011); and (iii) genes associated with laterality, such as *PCDH11X/PCDH11Y* (Williams et al., 2006). Recall that both brain size and laterality have long been thought to underlie our language-ready brain. But if we are right, language-readiness cannot be understood in the absence of a detailed characterization of the shape of the human head. Accordingly, we have done extensive text mining and database search in order to gain a better understanding of the genes that could account for the observed changes in AMH skull and brain, and eventually for our language-readiness. We have sought to define a gene candidate set on the basis of the following considerations, all ultimately related to globularity and language:

(1) The candidate has experienced some evolutionary change in our clade, and ideally, in our species after the split from Neanderthals/Denisovans. The type of change we have in mind concerns non-synonymous single-nucleotide polymorphisms (SNPs), insertions–deletions (InDels), changes in its expression level/pattern, new splicing variants, etc.
(2) The candidate plays some role in brain growth, regionalization, and/or neural interconnection, and specifically, in the development of the thalamus and its connection to the cortex.
(3) A mutation affecting the candidate gives rise to a clinical condition in which language, or cognitive properties often associated with language, is known to be impaired.
(4) The candidate is a candidate gene for craniosynostosis or some other similar condition at the phenotypic level such as cleidocranial dysplasia. This is clearly relevant to our hypothesis as the timing of suture closures clearly interacts with brain growth.

It stands to reason that these four considerations are but points of entry into the molecular basis of globularity. We do not for a moment believe that we have reached an exhaustive list, but we think that the genes we report on in this section can serve as a solid basis to characterize the interactome that underlies the language-ready brain. Ultimately, the candidate set as a whole serves as an additional testing ground for our hypothesis.

Concerning the methodological approach, our modus operandi was the following:

(1) We first searched the literature for candidate genes for craniosynostosis and related diseases in which cranial sutures become prematurely fixed or are not fixed at the proper time during the ontogeny. We also searched for genes that have been related to craniofacial development, or more generally, skull morphology. We compiled a tentative list of putative genes related to these phenotypes.
(2) We searched the literature for genes that play some role in the development of the thalamus, during fetal development or, preferably, after birth, given the timing of the globularization phase reported on in Section “Globularity.” We also compiled a tentative list of candidate genes.
(3) We matched both lists and suggested a tentative list of candidate genes to be used for the phylogenetic analysis.
(4) We searched the Neanderthal and Denisovan genomes for changes at the sequence level in any of our candidates compared to the human homologs. We explored the Neanderthal genome using both the Ensembl^1^ and the UCSC^2^ Genome Browsers. We also relied on the paper and the raw material delivered by Green et al. (2010). Concerning the Denisovan genome, we made use of the material provided by Meyer et al. (2012), including the valuable information provided in the supplementary materials.
(5) We also looked if our candidates have experienced some change in their expression patterns and splicing profiles. We mostly relied on the comparative analyses of the human vs. primate transcriptomes performed by Konopka et al. (2012).
(6) We improved the functional analyses of our candidates *in silico*, looking for:
(a) their expression patterns at the brain level, both in the adult brain and during development both before and after birth. For the adult brain we made use of the microarray database of the Allen Brain Atlas^3^, which we visualized via the Brain Explorer^®^ 2 tool. For the developing brain we made used of the Prenatal LMD Microarray search engine^4^ and the Developmental Transcriptome browser^5^ of the Allen Brain Atlas.
(b) their interactome. We searched for protein–protein known and predicted interactions via the String 9.05 tool^6^. String 9.05 predicts direct (i.e., physical) and indirect (i.e., functional) associations between proteins that derive from four sources: genomic context, high-throughput experiments, conserved coexpression, and the knowledge we had previously gained from text mining. We also searched extensively the literature looking for functional links of interest between our candidates and with other genes related to brain development, skull development, and to language.
(c) the linguistic and cognitive deficits linked to their mutation. We extensively explored the existing literature about this issue via the PubMed browser. We also searched the OMIM database, which is maintained by the National Center for Biotechnology Information^7^.
(7) We tried to refine our search for candidate genes by testing if some of our candidates’ partners within their respective interactomes (as provided by String 9.05) satisfy some of our four criteria. As before, we were mostly interested in genes that have experienced some evolutionary change in our species.
(2) We tried to confirm the hypothesis that some or all of our candidates played some important role also in the emergence of language properties by determining if some functional link(s) exist(s) between (some of) them and any of the “language genes” already identified in the literature. For achieving this we tried to determine if:
(a) they functionally interact at some level. We made use of String 9.05 and performed multiple searches that include the whole set of our candidates and the whole set of language-related genes compiled by Benítez Burraco (2009). We wanted to see if our candidate’s network(s) interact(s) with those of other language-related genes, and paradigmatically with that of *FOXP2*.
(b) any of our candidates and any of these “language genes” belong to the same functional module(s) as proposed by Konopka et al. (2012). In this case, we focused especially on *FOXP2* and its functional targets, both upstream and downstream the gene within its regulatory network.

Based on these, we arrive at the following tentative candidate set:

*USF1*, *RUNX2*, *DLX1*, *DLX2*, *DLX5*, *DLX6*, *BMP2*, *BMP7*, *DISP1*.

Below we briefly describe the biological relevance of each gene in the context of our hypothesis. As a general remark, though, let us make clear that we are not suggesting that all these genes were selected for allowing the emergence of the language-ready brain. Instead, as they are functionally connected, we expect that some evolutionary change occurred in one (or some) of them, which would have affected the whole network they are engaged in.

(1) *USF1*. This gene encodes a transcription factor involved in regulating synaptic plasticity, neuronal survival and differentiation (Tabuchi et al., 2002; Steiger et al., 2004), but also lipid metabolism (Lee et al., 2006). Together with other related transcription factors, this gene might be involved in the basal transcriptional machinery of *APOE* (Salero et al., 2003). This latter gene has been consistently related to some of the metabolic changes that allowed bigger brains, and eventually enhanced cognitive capacities, to evolve within hominins (Bufill and Carbonell, 2006). Interestingly, some polymorphisms of *USF1* have been related to Alzheimer’s disease (Isotalo et al., 2012). Moreover, USF1 binds to the promoter of *FMR1* (Kumari and Usdin, 2001). The hypermethylation (i.e., epigenetic silencing) of this promoter gives rise to fragile X syndrome, an extensively studied cognitive disorder (O’Donnell and Warren, 2002). Additionally, according to String 9.05, two putative partners of USF1 are CTNNB1 (interactors of this gene have been related to autism; O’Roak et al., 2012) and HRAS (the locus of the gene, 11p15, is a locus for dyslexia; the gene has also been linked to autism and encodes a GTPase involved in neural growth and differentiation, long-term potentiation, and synaptic plasticity; Comings et al., 1996). Another functional partner of USF1 is GTF2I (Roy et al., 1997). *GTF2I* has been related to cognitive disabilities and also to craniofacial abnormalities together with two other genes of its family also located in the 7q11.23 region in Williams syndrome (Morris et al., 2003; Tassabehji et al., 2005). Interestingly, GTF2I represses *RUNX2* (Lazebnik et al., 2009), one of our candidate genes (more on this gene below). Importantly, the regulatory region of *USF1* has undergone 30 fixed or high frequency changes after our split from Denisovans (Meyer et al., 2012).
(2) *RUNX2*. It controls different aspects of the morphology of the upper body and the cranium: closure of cranial sutures, clavicle development, rib cage formation, and dental growth (Stein et al., 2004). It is known to cause cleidocranial dysplasia (Yoshida et al., 2003), which is characterized by delayed closure of cranial sutures, hypoplastic or aplastic clavicles, a bell-shaped rib cage, and dental abnormalities (Mundlos et al., 1997). As a general rule, one can say that the greater amount of RUNX2 in the brain, the shorter interval time in which skull sutures remain open. Additionally, the gene appears to play an important role at the brain level. Significantly, it is highly expressed throughout the thalamus (Reale et al., 2013) and is involved in the control of rhythmic behavior (Reale et al., 2013). It is significantly downregulated in the hippocampus of bipolars and seems to play some important role in the development of GABAergic neurons in this area (Benes et al., 2007). RUNX2 indirectly interacts with β-catenin. In fact, β-catenin, RUNX2, and DLX1, DLX2 (two of our candidate genes) are key components of the GAD67 regulatory network, which is important for the normal development of GABAergic neurons within the hippocampus (Pleasure et al., 2000).

There is solid evidence of a selective sweep in *RUNX2* after our split from Neanderthals (Green et al., 2010). Interestingly, *RUNX2* is mentioned in Schlebusch et al. (2012), who, as part of their examination of the Khoe-San genome, performed a search for unusual stretches of high-frequency derived variants shared among extant population. [Due to their early divergence (Veeramah et al., 2012), signals of selection shared between Khoe-San and other populations offer a window into the evolutionary processes that occurred 100 kya, the critical period for the origin of AMH].

RUNX2 is stabilized by a protein called PIN1, to the extent that Pin1 mutations give also rise to cleidocranial dysplasia-like phenotypes in mice (Yoon et al., 2013). Interestingly, PIN1 regulates neuronal differentiation (Nakamura et al., 2012) and it is also involved in the onset of Alzheimer’s disease, influencing tau phosphorylation and amyloid precursor protein processing (Lonati et al., 2011; Arosio et al., 2012). [In the thalamus it is around birth when PIN1 expression levels change during development (as per the Human Brain Transcriptome database^8^)]. We believe that this can contribute to supporting the view that *RUNX2* modifications prompted some change(s) in brain development and not just in the development of the skull.

(3) *DLX1*. This gene controls skull morphology, thalamic development, and brain development and interconnectivity. In humans *DLX1*, along with *DLX2*, is expressed in neocortical GABAergic neurons (Letinic et al., 2002) and specifically regulates neuron differentiation in the ventral thalamus (Andrews et al., 2003; Jones and Rubenstein, 2004). It also contributes to connect thalamic nuclei with different neocortical domains. Mouse Dlx1/2(-/-) embryos (i.e., embryos in which both copies of the genes are knocked out) exhibit a shifted topography, even when regionalization defects in the thalamus or neocortex are not observed (Garel et al., 2002). This shift is first observed inside the basal ganglia, which develop abnormally (Garel et al., 2002). A modification in the expression pattern of transcription factors like *DLX1* in the forebrain can actually explain the species-specific programs for the generation of neocortical local circuit neurons. *Dlx1* deletion in mice results in reduced glutamatergic input to the hippocampus (Jones et al., 2011). Moreover, the less *Dlx1* (along with *Dlx2*) is expressed in the cortex, the fewer interneuron subtypes are generated and the more migration disturbances appear during brain development (Ghanem et al., 2008). Finally, *DLX1* seems to be downregulated in autists (Voineagu et al., 2011).
(4) *DLX2*. This gene is required for tooth and craniofacial development (Jeong et al., 2008; Gordon et al., 2010). Along with *Dlx1* it is expressed in neocortical GABAergic neurons, but also in the ventral thalamus (Jones and Rubenstein, 2004). Some parts of the ventral lateral geniculate nucleus of the thalamus derive from the prethalamic lineage expressing *Dlx2* (but also *Dlx5/6*; Jones and Rubenstein, 2004). As for *DLX1*, its mutations give rise to different anomalies in craniofacial, limb, and bone development (Kraus and Lufkin, 2006). Similarly, it has been linked to autism and psychosis (Liu et al., 2009). According to Johnson et al. (2009), *DLX1* and *DLX2* are differentially expressed across the brain. This differential expression has been further confirmed by microarray analysis, by qRT-PCR, and, in the case of *DLX1*, also by immunohistochemistry (Johnson et al., 2009). McKinsey et al. (2013) suggest that Dlx1 and Dlx2 control via Zfhx1b some important steps of neuronal proliferation within the cortex. Interestingly, when *Zfhx1b* is downregulated, “cells that ordinarily would become cortical interneurons appear to transform toward a subtype of GABAergic striatal interneurons” (p. 83). This suggests that whenever *DLX1* and/or *DLX2* are upregulated, more cortical neurons are expected to be generated (and vice versa). McKinsey et al. (2013) also posit an interesting link between mutations within *Zfhx1b* (and plausibly *Dlx1*/*DLx2* as well) and epileptic behavior in people affected by Mowat–Wilson syndrome. As is well-known, there is a pervasive link between epilepsy and language disorders, usually involving genes belonging to the *FOXP2* network (Pal, 2011). Moreover, Mowat–Wilson syndrome is characterized by speech delay, mental retardation, microcephaly, delayed motor development, and what may perhaps be an archaic facial phenotype, to judge from the following description in Adam et al. (2006): “All [patients] had a characteristic facial feature of a prominent nasal tip with the columella extending below the ala nasi. Other common facial features included cupped ears with fleshy, upturned lobules, deep-set eyes, hypertelorism, medially flared and broad eyebrows, and pointed chin.”
(5) *DLX5/DLX6*. These genes encode bone morphogenetic factors that control different steps of skull development, but also of brain development (Kraus and Lufkin, 2006; Wang et al., 2010). As is true of other DLX factors, *DLX5* is seemingly involved in the regulation of the migration and differentiation of precursor cells that give rise to GABAergic neurons in the forebrain. Specifically, *DLX5* can contribute to identify different interneuron subpopulations in the adult neocortex (Cobos et al., 2006). *Dlx5* also exhibits restricted expression in mouse prethalamus (Jones and Rubenstein, 2004), plausibly playing some relevant role in thalamic development. In an autistic proband, Poitras et al. (2010) report a mutation in an ultraconserved *cis*-regulatory element of *DLX5*/*DLX6* (known as I56i and also a binding site for GTF2I) that affects neurons that are tangentially migrating to the cortex. Reduced activity is also observed in GABAergic interneurons of the adult somatosensory cortex. A link between *DLX5* and autism has also been suggested by other authors (e.g., Nakashima et al., 2010). Another cis-regulatory element inside *DLX5*, namely I56ii, is active in “GABAergic projection neurons that may derive from progenitors found in the ventral LGE [lateral ganglionic eminence] and then migrate tangentially following a dorsal-to-ventral route before they finally settle down between the SVZ [subventricular zone] and the globus pallidus in the deep mantle of the MGE [medial ganglionic eminence]” (Ghanem et al., 2008, p. 423). This means that I56ii marks a subgroup of striatal projection neurons at least in the early stages of development. It may be worth noting at this point that a growing number of authors implicate the striatum as a key component of language (e.g., Ullman, 2001; Lieberman, 2002). Significantly, *Dlx5* and *Foxp2* are expressed in the same intercalated cell masses of the amygdala in rats and non-human primates, and in almost the same neuronal populations of the striatum (Kaoru et al., 2010). Moreover, mutations on *DLX5* and *DLX6* give rise to hand and foot malformations, intellectual disability, craniofacial anomalies, and hearing loss (Kraus and Lufkin, 2006; Brown et al., 2010; Shamseldin et al., 2012). Importantly, DLX5 regulate the expression of *RUNX2* (Jang et al., 2011). As we pointed out above, GTF2I regulates in turn the expression of both *DLX5* and *DLX6*, and interacts as well with USF1. According to String 9.05 one of DLX5 partners within its network could be MECP2, the main candidate for Rett syndrome (Amir et al., 1999). Rett syndrome is a neurodegenerative condition in which language loss, problems for motor coordination, microcephaly, and autistic behavior are prominent symptoms (Uchino et al., 2001; Veenstra-VanderWeele and Cook, 2004). Finally, in mice Foxp2 controls the expression of both *Dlx5* and *Dlx6* via *Shhrs*, a non-coding RNA highly specific to the ganglionic eminences (Vernes et al., 2011).
(6) *BMP2*. This gene encodes a bone morphogenetic protein that plays an important role in skull development: human mesenchymal cells in the primary sutures of the skull exhibit robust responses to BMP2; the osteogenic effect of BMP2 transforms muscle into bone (Dwivedi et al., 2012). Additionally, BMP2 plays some relevant role during brain morphogenesis. For instance, normal neurogenesis in the ganglionic eminences and correct cortical neurogenesis depend on the transcriptionally based regulation of BMP2/4 signaling by some histone deacetylases (Shakèd et al., 2008). BMP2 has also been reported to be involved in the survival and differentiation of GABAergic neurons and dopaminergic neurons in the embryonic brain, and also in promoting generation of astrocytes (Shakèd et al., 2008). Finally, BMP2 can affect neural migration and/or cell pattern formation in different brain areas via PTEN and/or β-catenin. For instance, it inhibits PTEN protein degradation, at least in some pathological/experimental conditions (Waite and Eng, 2003). According to Beck and Carethers (2007) BMP2 could inhibit *PTEN* expression as well via the RAS/ERK pathway. Moreover, BMP2 interacts with β-catenin, acting synergistically together with Wnt proteins for antagonizing the sensory fate-inducing activity of Wnt/β-catenin. A consequence of this is that cell differentiation in the neural crest is suppressed (Kleber et al., 2005). Importantly, in mice *Bmp2* is expressed in the postnatal thalamus in a nucleus-specific fashion, suggesting that it plays some role in the postnatal thalamus unrelated to their known role in developmental patterning (Yuge et al., 2011). Although mutations in *BMP2* are more frequently linked to osteoporosis (Styrkarsdottir et al., 2003) and bone formation diseases, like brachydactyly (Dathe et al., 2009), the mutation of *PTEN* gives rise to an autism spectrum disorder that also encompasses macrocephaly (Butler et al., 2005). In affected people, language acquisition is delayed and attention deficit hyperactivity disorder (ADHD) symptoms are also commonly observed (Naqvi et al., 2000). Moreover, PTEN regulates neural migration and cell pattern formation in different brain areas, particularly in the cerebellum (Marino et al., 2002).

In mice Bmp2 (and also Bmp7) upregulates *Dlx1*, *Dlx2*, *Dlx5*, and *Runx2* (Bustos-Valenzuela et al., 2011). It is also worth noting that during tooth development Wnt5a increases the expression of *DLX1*, *DLX2*, and *RUNX2* mRNA, suggesting a functional link among them (Peng et al., 2010). Among the BMP2 partners, as predicted by String 9.05, we also find *CTNNB1* (as in the case of USF1), as well as *SHH*, a gene controlling brain size that is one candidate for microcephaly and has been positively selected in our clade (Dorus et al., 2004). According to String 9.05 DLX2 is a SHH partner as well. It is also a partner of FGF8 [*FGF8* is one of FOXP2 as targets (Spiteri et al., 2007)], a protein involved in the regionalization of brain tissues in mammals (Fukuchi-Shimogori and Grove, 2001)], and of SMAD9 [the locus of the gene, AUTS3, is linked to autism (Smith et al., 2002); MAD proteins usually regulate cell proliferation and differentiation (Massague, 1996)].

(7) *BMP7*. Like *BMP2*, this gene encodes a bone morphogenetic factor (Ozkaynak et al., 1990). Much like *BMP2*, it plays a main role in osteogenesis (Cheng et al. (2003), but also pivotal roles in skull and brain development (Segklia et al., 2012), including the thalamus (Yuge et al., 2011). Mutations in this gene give rise to eye anomalies, deafness, scoliosis, cleft palate and developmental delay, and even learning disabilities (Wyatt et al., 2010). As we pointed out above, there seems to be a close functional link between BMP7 (and BMP2) and RUNX2, DLX1, and DLX2.
(8) *DISP1*. This gene is a key component of the SHH signaling network, which plays a key role in thalamic development (Nakagawa and Shimogori, 2012). *DISP1* has experienced positive selection in modern humans that resulted in a change V/M in the protein (Green et al., 2010).

A close examination of Konopka et al. (2012) confirms that all our candidates seem to be interconnected to some level. For instance, *BMP2* and *USF1* belong to the same module (labeled “darkviolet” in Konopka et al., 2012). Modules like this one result from a coexpression network analysis that is based upon exons rather than whole genes and that was performed to “uncover an enrichment of gene coexpression patterns based on alternative splicing” (p. 608), whereas *DLX1* and *BMP7* plausibly interact strongly within module olivedrab3. Moreover, *RUNX2*, *DLX2*, *DLX5*, and *DLX6* strongly interact within module palegreen1. Interestingly, both *DLX1* and *RUNX2* are highly connected to other genes belonging to the module lavenderblush1.

Also according to the data generated by Konopka et al. (2012), all our candidates have experienced changes in their expression levels and/or splicing patterns and/or interconnection patterns compared to those of chimps and rhesus. For instance, *USF1* and *BMP2* have quite increased their connectivity within the module olivedrab2, while *DLX1* have reduced its connectivity within this module compared to that of chimps and rhesus. Olivedrab2 is an important module within Konopka et al.’s (2012) analysis, as many of the genes comprising it have increased their connectivity in humans and their connectivity patterns are also less conserved than in other primates. Moreover, *DLX1* is the only gene among our candidates that shows an enrichment of ELAVL2 binding motifs. ELAVL2 is a splicing factor that interacts with different microRNAs to regulate cortical neurogenesis via derepression of *Foxg1* (Shibata et al., 2011). (*FOXG1* mutations in humans lead to a syndrome of microcephaly and social and language impairment; Kortüm et al., 2011). According to Konopka et al. (2012) some of the changes in the splicing patterns observed in the genes belonging to this olivedrab2 module could be explained by the evolutionary modification in humans of the expression pattern of this regulatory factor. Interestingly, *FOXP2* and some of their functional partners (*CNTNAP2*, *CMIP*, and *ELP4*) belong to this olivedrab2 module. All of them have greatly increased their connectivity in humans compared to chimps and rhesus. Moreover, both *FOXP2* and *CNTNAP2* are enriched ELAVL2 target genes within this module.

On the whole, we think that our network could be primarily related to the specification, migration and interconnection of GABAergic neurons within the forebrain, to skull morphogenesis and to thalamic development. Aberrant development of GABAergic interneurons has been linked to several conditions, as autism, epilepsy, Rett syndrome, and schizophrenia (e.g., Di Cristo, 2007). As one may expect given the general cognitive character of these diseases, language is known to be impaired in most of these conditions (Uchino et al., 2001; Veenstra-VanderWeele and Cook, 2004; Tager-Flusberg et al., 2005; Radanovic et al., 2013). If we consider other members of their interactomes (for instance, PTEN, SHH, ELAVL2, FOXG1, etc.) this network could be involved in the control of brain size as well. Eventually, some functional link exists with networks that are important for language, paradigmatically that of FOXP2, of which some components have also been positively selected in our clade. In some cases, differences exist specifically between the AMHs and Denisovan proteins, as *CNTNAP2* exemplifies (Krause et al., 2007; Meyer et al., 2012). [Links between our core network members and the FOXP2 network are further reinforced by genes such as *SIRT1*, which has been linked to Alzheimer (Chang and Guarente, 2013) via *RUNX2* (Shakibaei et al., 2012; Srivastava et al., 2012). On globularity and Alzheimer, see also Bruner and Jacobs (2013)].

In addition to the candidate genes discussed so far, three more genes suggest themselves in the context of our hypothesis.

The first one is *MEF2A*. According to Somel et al. (2013), the 50–100 kb region upstream the gene shows an indication of recent positive selection in AMHs. Considering the role played by the gene at the brain level, a change in *MEF2A* expression could have potentially resulted in, or contributed to, the delayed peak expression and the increase in the overall mRNA abundance of synaptic genes that is characteristic of the prefrontal cortex of modern humans. Consequently, although Neanderthals had brains that were larger than modern humans’, the cortical synaptic development in them may have been faster (Liu et al., 2012). Our search has revealed that the highest levels of *MEF2A* RNA are detected in the thalamus around birth (as per the Human Brain Transcriptome database^9^). Additionally, this gene functionally interacts with some of the components of our network, at least outside the brain. For instance, in the cardiac muscle MEF2A binds *USF1* and *USF2* (Moore et al., 2003). In turn, overexpression of the USF proteins in myocytes significantly reduces the functional interaction between MEF2A and some of its functional partners (Moore et al., 2003). Moreover, in zebrafish *mef2a* expression can be activated by bmp2 signaling in neonatal cardiomyocytes to the extent that exogenous mef2a is sufficient to rescue *bmp2* mutants (Wang et al., 2007). Finally, SIRT1 activation by resveratrol also affects a MAPK5/MEF2A dependent signaling pathway (Gracia-Sancho et al., 2010).

The second gene of interest is *TSC1*. According to Normand et al. (2013), *Tsc1* deletion in the developing thalamus disrupts thalamocortical circuitry, neural function, and behavior. Mutations on this gene give rise to tuberous sclerosis, a condition that usually entails learning difficulties that affects language growth (de Vries et al., 2009). These outcomes reinforces the link between thalamic development, thalamocortical networks, autism, and epilepsy, a link characteristic of many language disorders. [Thalamic volume is reduced in high-functioning autists (Tsatsanis et al., 2003); moreover, the thalamus is both anatomically and functionally underconnected with different cortical regions in people with autism spectrum disorders (Nair et al., 2013)].

The third gene we would like to mention at this point is *OTX2*. We were led to this gene via DLX genes. According to Ma (2011), in zebrafish dlx genes are important for parvalbumin-positive GABAergic neuron development. If correct, this provides another link between our candidate gene set and FOXP2, given the convergent differential regulation of parvalbumin in the brains of vocal learners argued for Hara et al. (2012) and the role FOXP2 plays in the context of auditory–motor association learning (Kurt et al., 2012). Parvalbumin-positive cells are critically involved in cross-modal plasticity (Desgent and Ptito, 2012), and this is where *OTX2* comes into play. According to Sugiyama et al. (2009) Otx2 homeoprotein is an essential morphogen for embryonic head formation and is reused later in life as a “messenger” for critical period plasticity. Moreover, in the domain of vision, it “is stimulated by visual experience to propagate into the visual cortex, where it is internalized by GABAergic interneurons, especially parvalbumin-positive cells” (p. 69). In the same vein, Omodei et al. (2008) suggest that a link exists between OTX2, dopaminergic neurons within different subcortical areas, motor and sensorimotor behaviors, and eventually, Parkinson disease (a condition in which some aspects of language are disordered; Grossman, 1999). Not surprisingly, there seems to be a functional link as well between this hub *OTX2* gene and our network. In particular, the interaction between BMP7 and OTX2 is important for the development of different brain regions: at least, the mid- and hindbrain (Tilleman et al., 2010) and the neuroepithelium (von Frowein et al., 2006; Müller et al., 2007). Moreover, the protease SPC7 cleaves the pro-BMP7 to release the corresponding active protein; in turn, *SPC7* knockdown is claimed to reduce the expression of *OTX2* in the anterior brain (Senturker et al., 2012). Additionally, both *DLX1* and *OTX2* are regionally restricted brain genes, important for the first stages of brain development (Yamamoto and Vernier, 2011). In fact, *OTX2* is one of the genes involved in early thalamic development, and also a component of the SHH signaling pathway that is a principal requirement for cell fate specification during thalamic development (Scholpp and Lumsden, 2010). Hemizygotic people have learning problems that impair language acquisition (homocygotic double mutants die early during development; Ragge et al., 2005). Finally, *OTX2* has been proposed as a candidate for some psychiatric disorders, particularly, bipolar disorder (Sabunciyan et al., 2007), in which some components of language processing are impaired.

**FIGURE 3:**
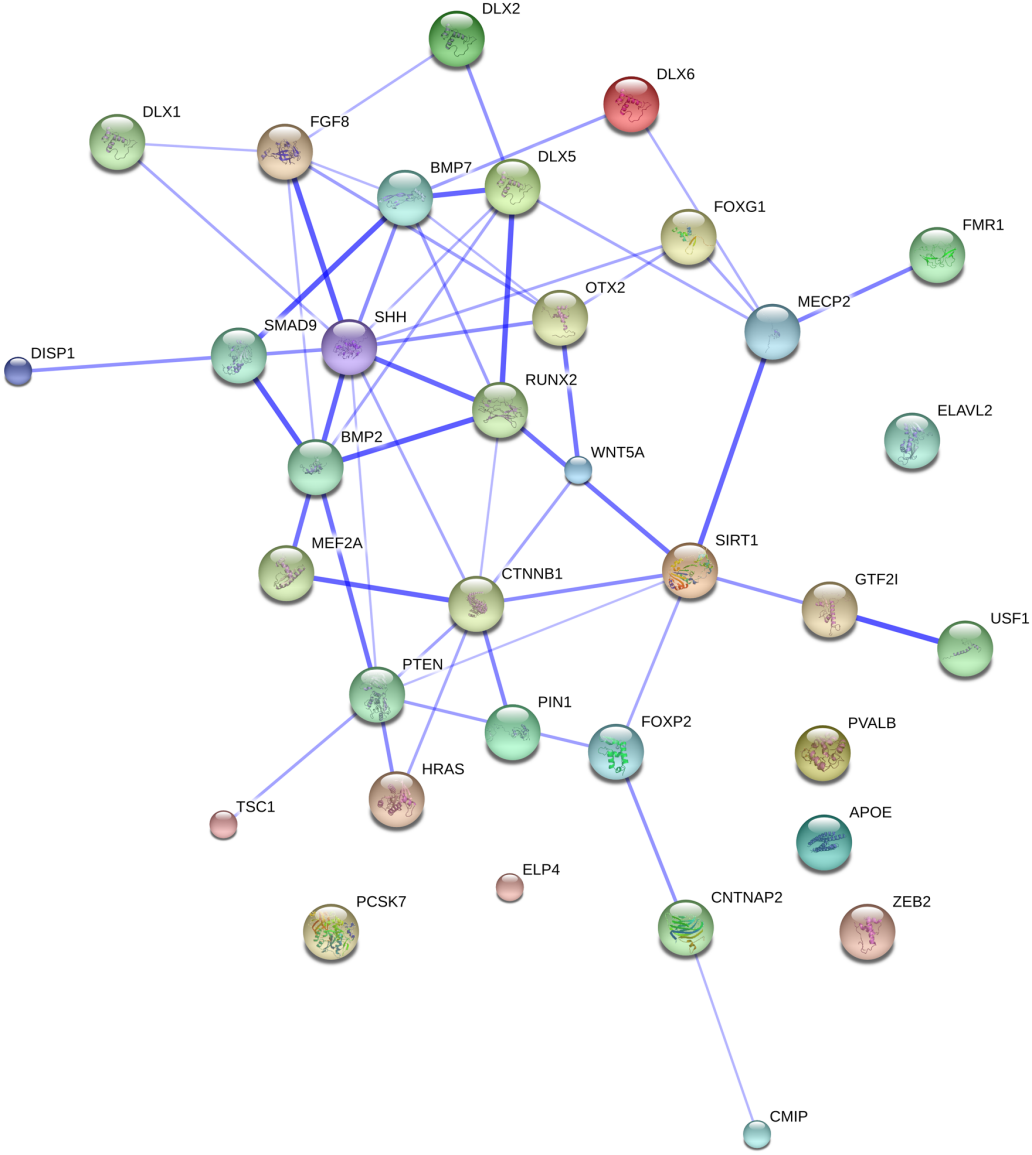
**The whole set of proteins encoded by the candidates genes for the language-ready brain.** The network was generated by String 9.05, a database of known and predicted protein interactions, either physical or functional (Szklarczyk et al., 2011). The medium confidence value was 0.04. Nodes representing the proteins encompassing the network are colored randomly. In this confidence view, stronger associations between proteins are represented by thicker lines. SPC7 and ZFHX1B are spelled according to their official symbol (PCSK7 and ZEB2, respectively).

The network displayed in **Figure 3**, generated via String 9.05, summarizes all our findings in a graphic fashion.

Let us stress that the genes identified in this section are not intended to exhaust the factors entering into globularity and the formation and maintenance of language-ready brain, but we hope that they can serve as solid candidates in the future characterization of these aspects of the modern human phenotype, together with the FOXP2 interactome and the genes that contribute to achieving a brain size like that characteristic of our species.

It stands to reason that as new information about each gene becomes available, our candidate gene set will expand. To give but one example of this, as this article was under review, the most complete sequence to date of a Neanderthal genome was released (Prüfer et al., 2014). This is likely to be a rich source of information about our candidate gene set, once all the search tools become available for it. Already now, the supplementary material of Prüfer et al. (2014) contains relevant information. Prüfer et al. (2014) highlight a highly disruptive intergenic change near *CITED2* that is 99% derived in modern humans and ancestral in both Altai Neanderthal and Denisovan. Importantly, *CITED2* is a regulatory target of FOXP2 (Nelson et al., 2013). As we have reviewed above, we expect that our network is linked at some level to the FOXP2 network. And in fact, according to the HBT database, *CITED2* is highly expressed in the mediodorsal nucleus of the thalamus. Actually, from the early childhood forward this is the brain area where the gene is most expressed. Additionally, Cited2 interacts with Lhx2 (Glenn and Maurer, 1999), a transcription factor that controls thalamocortical axonal guidance by specific regulation of Robo1 and Robo2 receptors (Marcos-Mondéjar et al., 2012). Interestingly, *ROBO1* is one of the best-known candidate genes for dyslexia (Hannula-Jouppi et al., 2005), suggesting a link to some aspects of language already. Furthermore, both *Cited2* and *Runx2* are regulated by TGF (Luo et al., 2005), again suggesting another functional link between both our network and the FOXP2 network.

## OUTLOOK

Much as we began this article by saying that our emphasis on globularity takes us away from the more standard anatomical characterization of the language-ready brain in terms of laterality, or sheer brain size, the hypothesis put forth here definitely downplays the role of standard language-brain areas: Broca’s region and Wernicke’s territory. We certainly recognize the linguistic role of these areas, or, more accurately, of the networks for which these regions serve as hubs. But we believe that they play a much more significant role at the level of externalization, an aspect of language that we have kept distinct from our focus here (cf. Hypothesis and Overview). We agree with Fedorenko et al. (2010) and the works cited in that study that high-level linguistic processing is accomplished by the joint engagement of two functionally and computationally different brain systems: (i) the classic “language regions” on the lateral surfaces of left frontal and temporal lobes that appear to be quite functionally specialized for linguistic processing and (ii) the fronto-parietal network, a set of cortical regions that is engaged across a wide range of cognitive demands and that we have argued are crucially regulated by the thalamus. As Fedorenko et al. (2010) note, most past neuroimaging work on language processing has not explicitly distinguished between these two systems, especially in the frontal lobes, where subsets of each system reside side by side within the region referred to as “Broca’s area.” In addition, we believe that much work in neurolinguistics has unintentionally emphasized the externalization component of language, since morpho-phonology is perhaps the easiest aspect to single out linguistic tasks, even if the word “syntax” was said to be the target of the relevant works. In so doing, work on neuroimaging biased the results toward the Broca–Wernicke model, and all too quickly attributed “syntax” to Broca’s area (see Bornkessel-Schlesewsky and Schlesewsky, 2013 for a converging view). When properly re-assessed in light of what theoretical linguistics takes to be syntax as opposed to the externalization component (Berwick et al., 2013a), such works may well confirm Broca’s initial intuition that these primarily pertained to the faculty of articulate language, although of course we expect that these areas eventually connect with the network envisaged here for the syntax–semantics interface. We hope that future work will elucidate the manner in which this connection takes place once the hypothesis put forth here is more firmly established in neurolinguistic circles.

To repeat comments we made in Section “Hypothesis and Overview,” this is not to deny the importance of morpho-syntax or externalization in the context of the linguistic brain. These are important aspects of modern language, but we think it is useful to keep these aspects separate from those we have focused on here. Also, we do not mean to exclude that a globular brain had other consequences for cognition, besides those we discussed here. For instance, Lieberman (2011) suggests that a more globular brain case had important consequences for our phonetic inventory. We leave an investigation of such consequences for future work.

The present hypothesis has clear implications in the context of clinical linguistics as well. As we have shown in the previous sections, the emphasis laid on the regulatory role of the thalamus in the account proposed here makes numerous connections with the literature on cognitive and language disorders such as autism, schizophrenia, etc. that view them as disconnection syndromes (hyper- and/or hypo-connectivity), inhibition imbalance, and the like (Neul, 2011). Perhaps the clearest and most immediate connection of our hypothesis with the clinical linguistics literature comes from the in-depth neurolinguistic analysis of the language symptoms of a patient who incurred bilateral paramedian ischemic damage of the thalamus, carried out by De Witte et al. (2006). Their results – “a marked simplification of syntax, characterized in the patient by simple sentences and sentence fragments with a complete absence of embedded clauses” – strike us as consistent with the expectations that can be formed from the account put forth here.

Our hypothesis also generates testable predictions that could be met by a detailed investigation of situations where human skulls are artificially deformed, a practice attested in several cultures (Neumann, 1942) and perhaps even among Neanderthals (Trinkaus, 1982). Unfortunately, too little is known in this domain for us to discuss this topic further at this point. We also believe that ultimately our analysis must be reconciled with the variation we find at the population level regarding skull shape, although here too we find that too little is known at present for us to expand on this topic. We would like to stress that the globularity hypothesis makes crucial reference to an early postnatal developmental stage, at which point the skull is most globular (Vannucci et al., 2013). Adult deviations from this pattern, though significant, may not be the best data to use at first. Given that, as we have said, both shape and size parameters must be taken into account to characterize the language-ready brain, we think that our account would also benefit from a detailed investigation of the anatomical and cognitive consequences of microcephaly, although we will have to leave this topic for future research.

Outside the human range, our claim that different brain shapes entail wiring differences suggests that we should find these in comparing species that differ in brain shapes (for instance, dogs). At the moment, we do not know of studies that address this prediction of our hypothesis.

Our hypothesis will also benefit from future research on the ontogenetic and phylogenetic trajectories of the brain structures we have discussed. Insights into the gene expressions pertaining to these structures is likely to add significantly to the information we have already gathered.

To conclude this section, we would like to point out that it has not escaped our attention that if the hypothesis advanced in this work is on the right track, it makes it even more difficult to unravel the role natural selection may have played in the emergence of language, given the integrated nature of human head, well documented in Lieberman (2011). The human skull is a complex and highly integrated structure. Recent studies of the genetics of craniofacial variation reveal a very complex and multifactorial picture, with various factors such as locomotion, diet, and, of course, cognition being worth taking into account (Willmore et al., 2005; Burgio et al., 2009; Martínez-Abadías et al., 2009, 2012). These findings contrast with older ideas that posit much simpler developmental bases for variation in cranial morphology such as the growth of the brain, the face or the chondrocranium. Selective biases, as Lieberman (2008) points out, may have come from various domains, with brain growth being only one of them, making the adaptationist question one of those “we may never answer” (Lewontin, 1998).

## Conflict of Interest Statement

The authors declare that the research was conducted in the absence of any commercial or financial relationships that could be construed as a potential conflict of interest.

## Footnotes

1 http://projects.ensembl.org/neandertal/

2 http://genome.ucsc.edu/Neandertal/

3 http://human.brain-map.org/microarray/search

4 http://www.brainspan.org/lcm/search/index.html

5 http://www.brainspan.org/rnaseq/search/index.html

6 http://string-db.org/

7 http://www.ncbi.nlm.nih.gov/omim

8 http://hbatlas.org/

9 http://hbatlas.org/
